# Clinical, Metabolic, and Radiological Risk Factors in Individuals With Plantar Heel Pain From a South Indian Population: A Cross-Sectional Observational Study

**DOI:** 10.7759/cureus.42834

**Published:** 2023-08-02

**Authors:** Gopisankar Balaji, Vishal Reddy Duddukunta, Mohanakrishnan Jagadevan, Suresh Thappa, Deepak Barathi

**Affiliations:** 1 Orthopaedics, Jawaharlal Institute of Postgraduate Medical Education and Research, Pondicherry, IND; 2 Physiotherapy, Jawaharlal Institute of Postgraduate Medical Education and Research, Pondicherry, IND; 3 Radiodiagnosis, Jawaharlal Institute of Postgraduate Medical Education and Research, Pondicherry, IND

**Keywords:** radiography, risk factor, heel spur, fasciitis, heel

## Abstract

Background

One of the most common conditions seen in an orthopedic outpatient clinic is plantar heel pain (PHP). Studies analyzing various risk factors and their association with the development of PHP have been performed primarily in the Caucasian population, and no study has noted any association between the magnitude of various risk factors and their correlation to the severity of PHP. Hence, we performed a prospective cross-sectional observational study in a select South Indian population presenting with PHP to a tertiary care center.

Methods

All adult patients presenting to the orthopedic OPD between July 2019 and July 2020 were screened for unilateral PHP and were included after meeting the eligibility criteria. Age, sex, body mass index (BMI), random blood sugar (RBS), uric acid, thyroid-stimulating hormone (TSH), and vitamin D3 were measured as demographic and metabolic parameters. Heel pad thickness, calcaneal spurs, and plantar fascial thickness were noted radiographically. Clinically, the wall-toe distance by weight bear lunge test of each foot was noted, and the severity was measured by the foot functional index (FFI).

Results

Among the 40 participants, the mean age was 44 (±10.9) years. The average BMI was 30.1 (27.02-32.95). No significant association was noted between the biochemical parameters and the occurrence of PHP. The plantar fascial thickness (PFT) and heel pad thickness (HPT) were thicker than the asymptomatic foot by 1.01 (0.60 - 1.30) mm and 0.79 (0.4-1.7) mm, respectively, which was statistically significant (p<0.001). The heel cord length was found to be reduced by 0.86 (0.6-1) cms, which was statistically significant (p<0.001). The average FFI score was 123.07 (±15.57), and the FFI score percentage in individuals was 53.5% (±6.77). None of the above risk factors showed any significant correlation to the intensity of clinical symptoms measured by FFI (p>0.05).

Conclusion

Participants had a high BMI and a higher percentage of females. There was a significant increase in PFT and HPT thickness and a significant reduction in gastrocnemius flexibility when compared to the asymptomatic foot. There was no significant association between various clinical, metabolic, and radiological risk factors and the intensity of plantar fasciitis measured by FFI.

## Introduction

Plantar heel pain (PHP) is one of the most common causes of disability in the adult population [1]. Our understanding of the disease and its etiopathogenesis has evolved over time. We now shift from a previously described inflammatory fasciitis to a degenerative fasciopathy [2]. Similarly, our knowledge of risk factors contributing to PHP has also evolved during this time. However, risk factor association with PHP up until now has been only quoted from the literature [3,4], involving athletic individuals belonging to the Caucasian population. As concepts in the etiopathogenesis of PHP evolve, it is time to review and reassess various risk factors contributing to PHP in select demographic populations. This can help medical professionals provide the best patient care possible. A cross-sectional observational study was conducted to observe the incidence of metabolic, radiological, and clinical risk factors in individuals presenting with PHP from a tertiary care center in South India. Additionally, we analyzed if any correlation exists between the risk factors and the severity of plantar fasciitis measured by the foot functional index (FFI).

## Materials and methods

Study design

The trial was designed as a cross-sectional observational study. Prior to clinical commencement, the trial was registered with the institutional ethics committee of JIPMER (JIP/IEC/2019/067) and the Clinical Trial Registry of India (CTRI/2019/12/022250).

Setting and participants

Participants for the study were recruited from July 2019 to July 2020 from the orthopedic outpatient department (OPD) of a tertiary care center in South India after satisfying the selection criteria. Patients above 18 years presenting to the orthopedic OPD were screened for unilateral plantar heel pain. Following this, they were excluded if they had received previous treatment for PHP, had previous heel trauma or surgery, history of foot infection or osteomyelitis, an associated neurological disorder affecting foot function, or had a history of inflammatory arthropathy. Participants who met the above eligibility criteria were recruited into the trial after obtaining consent.

Variables and outcome measures

Age, sex, body mass index (BMI), affected foot, and type of footwear used (barefoot vs. footwear), average duration spent standing in a day (less than 8 hours vs. greater than 8 hours), and gross morphology of foot (noted as normal vs. flat vs. high arched) were measured as patient sociodemographic details. Key metabolic parameters analyzed at enrolment were random blood sugar (RBS), uric acid, thyroid stimulating hormone (TSH), and vitamin D3. Clinically, the wall-toe distance of each foot was calculated using the weight-bearing ankle lunge test to assess the Achilles tendon tightness. Radiographic measurements included measuring heel pad thickness and the presence of calcaneal spurs in lateral weight-bearing X-rays. Furthermore, plantar fascia thickness and heel pad thickness were calculated using ultrasonographic techniques. Clinical outcome was measured using the foot function index (FFI) questionnaire.

Data measurement and quantitative variables

Participants were enrolled based on convenient sampling. Following enrollment, demographic, clinical, hormonal, metabolic, radiological, and clinical outcomes measured by FFI pertaining to plantar fasciitis were collected by the researchers.

Key hormonal parameters were categorized as follows for statistical analysis and to increase the external validity of the results. Random blood sugar (RBS) was categorized into two groups, group A (normal RBS levels) involving patients with RBS <200 mg/dl and Group B (high RBS levels) with RBS ≥200 mg/dl. Uric acid was categorized into two groups, group A (normal uric acid levels) involves patients with uric acid <7 mg/dl, and Group B (high uric acid levels) with uric acid ≥7 mg/dl. Thyroid-stimulating hormone (TSH) was categorized into two groups, group A (normal TSH levels) involving patients with TSH ≤5 µIU/ml and group B (high TSH levels) with TSH >5 µIU/ml. Vitamin D3 was categorized into two groups, Group A (low vitD3 levels) involves patients with vitamin D3 <20 ng/ml, and Group B (normal vitD3 levels) with vitamin D3 ≥20 ng/ml.

Statistical analysis

The data were analyzed using SPSS Statistics software (Version 19.0, IBM Corp., Armonk, NY). Continuous variables were expressed as a measure of their means (± standard deviation) and medians (25-75 quartiles). Dichotomous variables were expressed as frequencies. Depending upon the normality of distribution, the significance of the difference in risk factors between symptomatic and asymptomatic foot was calculated using paired-t test or Wilcoxon signed-rank test. The chi-square test or Fischer’s exact was used to note the significance of the difference in incidence for nominal variables. Bivariate correlation analysis was performed to note any significant association between the objectively measured risk factors and clinical outcome.

## Results

Demographic characteristics of participants

Forty participants were enrolled in the study, i.e., 40 feet with a normal opposite foot as a control in an individual, and statistical analysis was performed on the data collected.

The mean age of the participants was 44 (±10.9) years. The average BMI of the individuals in the study was 30.1 (27.02-32.95). Eighty percent (80%; n=32) of the participants were females. Sixty-seven point five percent (67.5%; n=27) of the individuals had plantar fasciitis in the left foot. Fifty-two percent (52%; n=21) of the participants were involved in activities that predisposed them to greater than eight hours of standing. The majority of the participants were non-smokers (77.5%, n=31) and non-alcoholics (85%, n=34). Only 55% (n=22) of the individuals wore footwear; the rest walked barefoot.

Outcomes of clinical, radiographic, and metabolic risk factors

Clinically, the weight-bearing lunge test difference between the normal and affected foot was 0.86 (0.6-1) cm, which was statistically significant (p<0.001). The average FFI score was 123.07 (±15.57), and the FFI score percentage in individuals was 53.5% (±6.77). The individual components of FFI averaged a score of 48.98 (±8.27), 60.15 (±7.74), and 13.95 (10-17) for the pain, disability, and activity limitation subcomponents, respectively.

The radiographic parameters measured between the affected and normal foot are depicted in Table 1.

**Table 1:** Summary of the clinical WBLT and radiographic parameters measured between the affected and normal foot WBLT: weight-bearing lunge test

Parameter measured	Affected foot	Asymptomatic foot	P value
Wall-toe distance on WBLT (cm)	13.45 (13-13.8)	14.31 (14-14.8)	<0.001
PFT measured on ultrasonography (mm)	5.12 (4.82-5.40)	4.11 (3.8-4.37)	<0.001
HPT measured on ultrasonography (mm)	14.99 (14.5 -15.5)	14.20 (13.3 – 14.8)	<0.001
HPT measured on X-rays (mm)	16.02(14.72-17.87)	15.38 (13.80-17.27)	0.006
Incidence of calcaneal spurs	47.5%	62.5%	0.76

**Figure 1:**
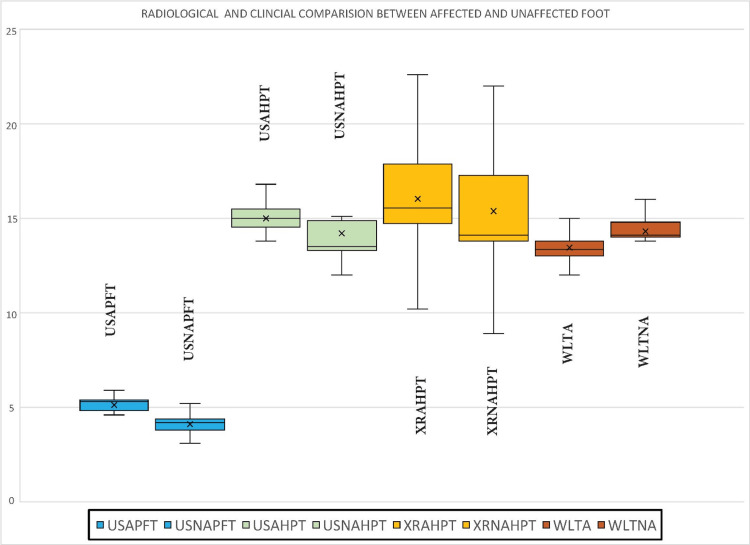
Box plot comparing the difference in: 1. Plantar fascia thickness (PFT) (mm) measured by ultrasound (blue box plot) (USAPFT; affected foot PFT measured by ultrasound, USNAPFT; unaffected foot PFT measured by ultrasound), 2. Heel pad thickness (HPT) (mm) measured by ultrasound (green box plot) and X-rays (yellow box plot) (USAHPT; affected foot HPT measured by ultrasound, USNAHPT; unaffected foot HPT measured by ultrasound, XRAHPT; affected foot HPT measured by X-rays, XRNAHPT; unaffected foot HPT measured by X-rays), 3. Weight-bearing lunge test (WBLT) (cm) measured by wall toe distance (orange box plot) (WLTA; wall toe distance of affected foot, WLTNA, wall toe distance of unaffected foot)

The incidence of a bony spur in the affected foot was 47.5% (n=19), which is not significant as a predictor in PHP (p=0.1). The mean difference in plantar fascial thickness (PFT) and heel pad thickness (HPT) measured by ultrasonography between the affected and normal foot was 1.01 (0.60 - 1.30) mm and 0.79 (0.4-1.7) mm, respectively. This difference in PFT and HPT measured on ultrasound was statistically significant (p<0.001). The difference in HPT measured on X-ray between the affected and normal foot was 0.64 (-0.45 - 1.47) and was statistically significant (p=0.006). A comparative box plot comparing the difference in HPT, PFT, and weight-bearing lunge test (WBLT) between the symptomatic and asymptomatic foot is depicted in Figure 1.

Analysis of the metabolic risk factors shows that only 17.5% (n=7) of the participants had high sugar (RBS >180mg/dl), 7.5% (n=3) of participants had high uric acid levels (uric acid >7 mg/dl), 25% (n=10) had low vitamin D3 levels (vit D3 levels < 20 ng/ml), and 15% (n=6) had high TSH levels (TSH > 5mIU/l). There was no significant association between the metabolic parameters toward the occurrence of PHP (p>0.05). Table 2 represents the data regarding sociodemographic, clinical, and metabolic risk factors.

**Table 2:** Summary of the FFI score, socio-demographic, and metabolic risk factors FFI: foot function index; TSH: thyroid-stimulating factor; RBS: random blood sugar

PARAMETER MEASURED	VARIABLES	N	FREQUENCY
SEX	Male	8	20%
	Female	32	80%
AFFECTED FOOT	Left	27	67.5%
	Right	13	32.5%
HOURS OF STANDING PER DAY	<8 hours	19	47.5%
	> 8 hours	21	52.5%
SMOKING	No smoking	1	77.5%
	Smoking present	9	22.5%
ALCOHOLISM	Non-alcoholic	34	85%
	Alcoholic	6	15%
FOOTWEAR	Barefoot	18	45%
	Footwear usage	22	55%
TSH	TSH ≤5 µIU/ml	34	85%
	TSH >5. µIU/ml	6	15%
VITAMIN D3	Vitamin D3 <20 ng/ml	10	25%
	Vitamin D3 ≥20 ng/ml	20	75%
RBS	RBS <200 mg/dl	33	82.5%
	RBS ≥200 mg/dl	7	17.5%
URIC ACID	Uric acid <7 mg/dl	37	92.5%
	Uric acid ≥7 mg/dl	3	7.5%

**Figure 2:**
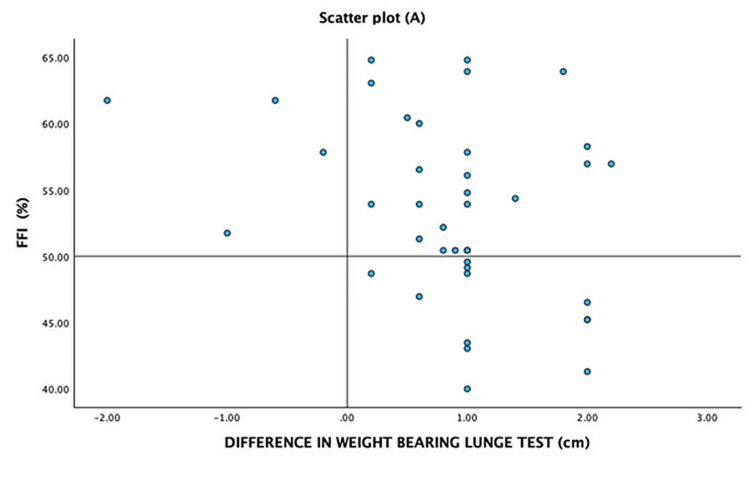
Scatter plot (A) – distribution of participants with respect to the difference in WBLT and severity of clinical symptoms measured by FFI WBLT: weight-bearing lunge test; FFI: foot functional index

**Figure 3:**
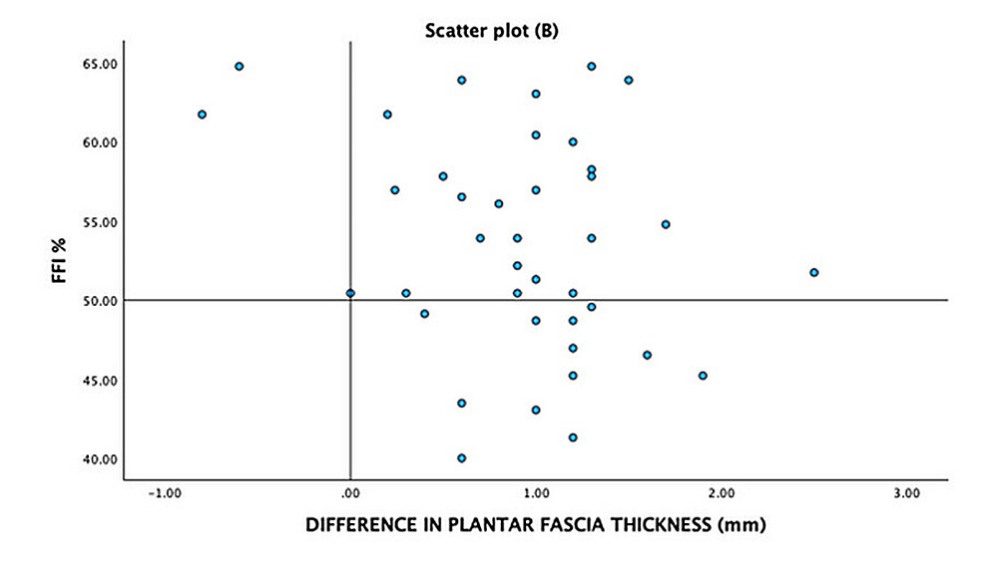
Scatter plot (B) – distribution of participants with respect to the difference in plantar fascial thickness and severity of clinical symptoms measured by FFI FFI: foot functional index

**Figure 4:**
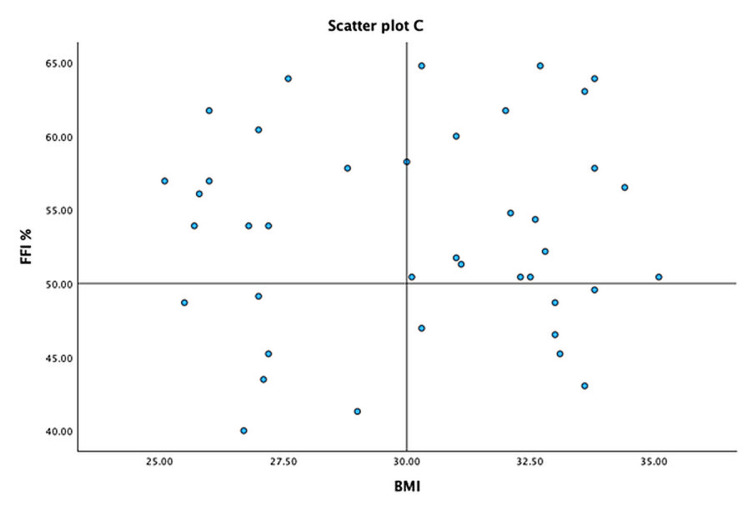
Scatter plot (C) – distribution of participants with respect to the BMI and severity of clinical symptoms measured by FFI FFI: foot functional index

To better understand the trends, we represent the severity of symptoms of PHP measured by FFI and the difference in WBLT, the difference in PFT, and BMI on a quadrant scatter plot -A (Figure 2), B (Figure 3), and C (Figure 4), respectively. We note that the majority of the participants with lower weight-bearing lunge distance and greater PFT on the affected foot had more severe foot symptoms as measured by FFI. However, the severity of the symptoms had no relation with the BMI of the patient, as seen by the diffuse scatter plot.

Ancillary analysis

To note if any risk factor could predict the severity of clinical symptoms, a bivariate correlation analysis between BMI, sex, hours of standing in a day, plantar fascial thickness, heel pad thickness, and weight-bearing lunge test to FFI score was performed. None of the above risk factors showed any significant correlation to the intensity of clinical symptoms measured by FFI (p>0.05).

## Discussion

PHP has a lifetime incidence of 1 in 10 people worldwide, and 90% of the patients have their symptoms resolved within 12 months, but there is a tendency for PHP to develop into a chronic pain condition [2]. Therefore, studies on risk factor analysis and their role in predicting clinical severity help in the prognostication and prevention of PHP.

Our study noted that the affected foot had a statistically significant increased plantar fascial and heel pad thickness, which is concordant with the studies performed by Aradhya et al. and Tsai et al. [5,6]. However, the incidence of a bony spur in our study was not a significant predictor of PHP. This lack of association is contradictory to the results obtained by Leeuwen et al. and McMillan et al. [4,7]. We also noted that the magnitude of PFT and HPT did not correlate with the severity of plantar fasciitis as measured by FFI. This is similar to that seen by Hansen et al. [8].

There is increasing evidence to state that gastrocnemius’s lack of flexibility is a potential contributor to developing PHP [9]. We assessed this clinically during enrolment via WBLT [10] and noted that the wall-toe distance in the affected leg was significantly less than that of the normal foot. This is in accordance with the results by Riddle et al. and Kibler et al. [11,12]. It is therefore advisable to screen the heel cord flexibility as a cause of PHP, as appropriately directed physical therapy intervention in improving the gastrocnemius flexibility would significantly alleviate the pain associated with PHP. Interestingly, however, WBLT didn’t have any correlation to the intensity of clinical symptoms measured by FFI.

Among the common metabolic risk factors noted, we did not note any significant association of these risk factors with the development of PHP. This is in concordance with the study by Yadav et al. [13], wherein they note that uric acid levels had no correlation with developing PHP. Our study is in concordance with that of Gariani et al. [14], who noted that diabetes as a risk factor doesn’t increase the incidence of PHP.

Higher BMI and female gender have a predilection to the development of PHP [4,5]. We were limited by the lack of a comparator group to provide any insight on the increased risk of developing PHP in relation to these risk factors. However, we noted that BMI, gender, and hours of standing in a day do not have any significant correlation to the severity of clinical symptoms measured by FFI. Hence, general physician bias and negative counseling in these subsets of patients should be avoided, as these factors cannot be managed acutely.

Many times, structural and morphological changes do not always reflect on the functional outcome measures [16]. We found a similar pattern of no significant correlation trends between objective parameters measuring various risk factors to the subjective functional disability in an individual. This strengthens the case put forth by Klein et al. that individuals with PHP seek treatment only for pain even though they do not exhibit functional setbacks [17]. This is true in acute and chronic conditions, and it is worth noting that even when the condition becomes chronic, individuals with PHP show no worsening of functional measures [17].

Due to coronavirus disease 2019 (COVID-19) restrictions during the enrollment of participants in this study, we acknowledge the limitation in the sample size and the power associated with the statistical analysis results.

Our study depicts the incidence of various risk factors in PHP in the South Indian population. Some risk factors validate trends from existing data from studies performed in different ethnic populations while others contradict them. However, one should note that these results are only representative of trends in the select demographic population, and further studies need to be conducted to ascertain any risk factor association definitively.

## Conclusions

In our study, we observed that the participants had a high BMI and a higher percentage of females. There was a significant increase in PFT and HPT thickness, and a significant reduction in gastrocnemius flexibility when compared to the asymptomatic foot. There was no association between the intensity of plantar heel pain measured by the foot functional index and the various clinical, metabolic, and radiographic risk factors.
